# Direct Observation of Monolayer MoS_2_ Prepared by CVD Using In-Situ Differential Reflectance Spectroscopy

**DOI:** 10.3390/nano9111640

**Published:** 2019-11-19

**Authors:** Yina Wang, Lei Zhang, Chenhui Su, Hang Xiao, Shanshan Lv, Faye Zhang, Qingmei Sui, Lei Jia, Mingshun Jiang

**Affiliations:** 1 School of Control Science and Engineering, Shandong University, Jinan 250061, China; ynwangsdu@163.com (Y.W.); suchenhui2010@163.com (C.S.); 15275139781@163.com (H.X.); zhangfaye@sdu.edu.cn (F.Z.); qmsui@sdu.edu.cn (Q.S.); jialei@sdu.edu.cn (L.J.); sdujiangmingshun@163.com (M.J.); 2 Institute of Marine Science and Technology, Shandong University, Qingdao 266237, China; sdulvshanshan@163.com

**Keywords:** in-situ differential reflectance spectroscopy, chemical vapor deposition, transition metal dichalcogenides

## Abstract

The in-situ observation is of great significance to the study of the growth mechanism and controllability of two-dimensional transition metal dichalcogenides (TMDCs). Here, the differential reflectance spectroscopy (DRS) was performed to monitor the growth of molybdenum disulfide (MoS_2_) on a SiO_2_/Si substrate prepared by chemical vapor deposition (CVD). A home-built in-situ DRS setup was applied to monitor the growth of MoS_2_ in-situ. The formation and evolution of monolayer MoS_2_ are revealed by differential reflectance (DR) spectra. The morphology, vibration mode, absorption characteristics and thickness of monolayer MoS_2_ have been confirmed by optical microscopy, Raman spectroscopy, ex-situ DR spectra, and atomic force microscopy (AFM) respectively. The results demonstrated that DRS was a powerful tool for in-situ observations and has great potential for growth mechanism and controllability of TMDCs prepared by CVD. To the best of the authors’ knowledge, it was the first report in which the CVD growth of two-dimensional TMDCs has been investigated in-situ by reflectance spectroscopy.

## 1. Introduction

Two-dimensional (2D) materials have attracted enormous attention in optoelectronic devices, flexible sensors, catalysis, and energy conversion due to their outstanding physical and chemical properties [1,2,3,4,5,6,7,8]. In particular, 2D transition metal dichalcogenides (TMDCs) have tunable band gaps, which make their application more likely in semiconductor optoelectronic devices than graphene [4]. The preparation of large-area and high-quality 2D thin film materials is the basis for their extensive development and application. The growth mechanism is not well understood yet, although graphene and MoS_2_ have been successfully synthesized at the wafer-level size [9,10]. The efforts about in-situ observation during growth are conducted to explore the growth mechanism and control the quality of 2D materials. However, it is quite challenging to investigate the growth process in-situ because the 2D materials normally require high temperature and a wide range of temperature variations.

There are various synthetic methods of 2D nanomaterials including micromechanical exfoliation, ion intercalation-assisted liquid exfoliation, molecular beam epitaxy (MBE), atomic layer deposition (ALD), and chemical vapor deposition (CVD) [11,12,13,14,15]. 2D materials with high-quality and high-efficiency can be synthesized by CVD, which is suitable for industrial large-scale preparation [14,16,17,18]. Therefore, CVD has been considered as a promising technology for the industrial preparation of 2D nanomaterials [19]. However, the requirement of high-temperature conditions and special gas environment limits the in-situ measurements, leading to difficulty for most characterization methods.

In recent reports, 2D materials were investigated by ex-situ characterized methods, such as optical microscopy, atomic force microscopy (AFM), and Raman spectroscopy [20,21,22,23]. Only a few studies focused on the in-situ investigation of 2D materials growth now. Chai and Wang studied the orientation and grain size of MoS_2_ flakes by transmission electron microscopy (TEM) [24]. However, this research reported the non-real-time characterization of MoS_2_ prepared by the thermolysis of (NH_4_)_2_MoS_4_. Leick et al. have used spectroscopic ellipsometry (SE) for in-situ study of thin film growth [25]. SE system requires a high structural stability which is difficult to develop based on a CVD system. The assembly of a SE in-situ characterization system is more difficult than differential reflectance spectroscopy (DRS). Sun et al. in 2017 introduced an in-situ study about molecular beam epitaxy (MBE) growth of MoS_2_ using DRS [26]. The results showed that DR spectra was sensitive to detect growth and optical properties of 2D materials. In 2019, Sun’s team also showed in-situ differential transmittance spectroscopy (DTS) of MoS_2_ grown by CVD on the sapphire substrate [27]. This was the first report about the in-situ characterization of layered TMDCs materials grown by CVD. However, the DTS is only applicable to transparent substrates. Molybdenum trioxide (MoO_3_) film was deposited on the observation window, and the growth information of MoS_2_ at late growth stage was shielded. In summary, the research about in-situ reflectance spectroscopy of 2D TMDCs materials grown by CVD remains to be further explored.

DRS is commonly used in the in-situ detection of thin film preparation as a result of its fast measurement speed and high sensitivity [28,29]. Fritz et al. developed a DRS system for on-line detection of organic film growth by combining DRS optical structure with vacuum system [29]. Castellanos-Gomez et al. studied the relationship between the thickness of TMDCs and exciton energy by ex-situ DRS [23]. Combined with the [26], DRS technology has become one of the most promising technologies for in-situ detection of 2D material prepared by CVD.

In this work, the CVD method was utilized to prepare MoS_2_ thin films on the Si substrate covered by 300 nm-thick SiO_2_. Optical microscopy, Raman spectroscopy, atomic force microscopy, and ex-situ DRS were applied to characterize samples offline. More importantly, in-situ DR spectra revealed the evolution in reflectivity and growth process during the preparation of MoS_2_. The results provide a detailed understanding of the growth evolution for 2D materials and emphasize that DRS is a powerful tool for the in-situ characterization of 2D TMDCs prepared by CVD.

## 2. Materials and Methods

### 2.1. Preparation of MoS_2_ by CVD

The atmospheric-pressure CVD was applied to prepare MoS_2_ samples in a two-zone tube furnace. A silicon substrate with 300 nm thick SiO_2_ was selected as the growth substrate. The substrate was ultrasonically cleaned in acetone, alcohol and DI water. Then it was dried by nitrogen gas gun. Finally, the substrate was placed face-up on a crucible boat and loaded into the temperature-zone ІІ of a tube furnace. 15 mg MoO_3_ power (>99.5%, Sigma-Aldrich, St. Louis, MO, USA) was placed upstream on the same crucible boat with the substrate. The distance between MoO_3_ and the substrate is approximately 6 cm. After weighing the sulfur powder (>99.5%, Sigma-Aldrich, St. Louis, MO, USA), a second crucible boat containing 1 g of sulfur was located in the temperature-zone І of the furnace. During the growth process, high-purity argon gas was injected with a flow rate of 40 sccm. It aimed at maintaining an inert atmospheric-pressure environment in a tube furnace and acted as the carrier gas. The tube furnace was heated up to 750 °C during 50 min. The temperature was maintained for 15 min before cooling down to room temperature.

### 2.2. Ex-Situ Characterization Experiment

Optical images were obtained using an optical microscope (GFM-550, Shanghai Guangmi Instrument Co., Ltd., Shanghai, China) to observe the morphology of MoS_2_ on the SiO_2_/Si substrate. Raman spectra were performed by a Raman microscope (Renishaw inVia, Gloucestershire, UK) with a laser excitation wavelength of 532 nm at the room temperature. The detection was carried out using a 2400 grooves mm^−1^ grating. The ex-situ DRS system was built on an optical platform. It consists of a tungsten light source, two converging mirrors and a detector. A spectrometer (QE Pro, Ocean Optics, Dunedin, FL, USA) as the detector was used to collect the reflected light. The ex-situ DR spectra were defined by: [30,31]

(1) $$\frac{\Delta R}{R}=\frac{R_{S}-R_{0}}{R_{0}},$$

where *R*_0_ and *R_S_* denote the reflected light intensity of the bare substrate and regions covered with MoS_2_, respectively. The thickness and surface topography of the sample were conducted by AFM (Dimension Icon, Bruker, Santa Barbara, CA, USA) in PeakForce tapping mode. We used an AFM cantilever (ScanAsyst-Air, Bruker, Santa Barbara, CA, USA) with a resonant frequency of approximately 75 kHz and a spring constant of approximately 0.4 N/m. All ex-situ characterization experiments were carried out at room temperature.

### 2.3. In-Situ DRS Experiment

**Figure 1:**
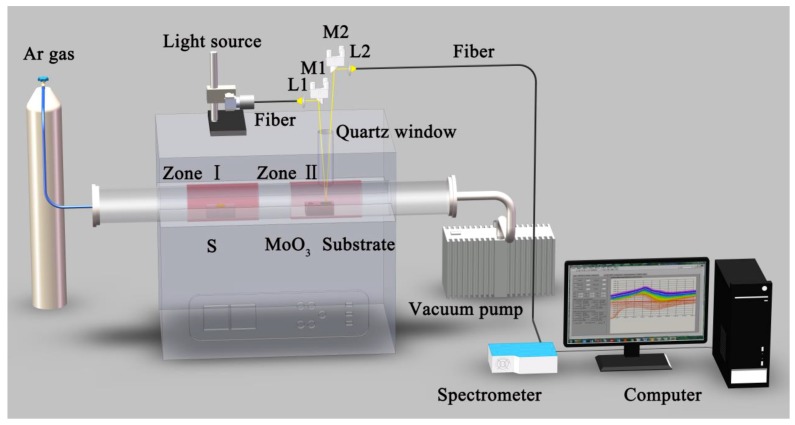
Scheme of the in-situ experiment device.

The in-situ experiment setup consists of CVD preparation equipment and the in-situ DRS equipment. This is a universal detector to in-situ detect the growth of 2D materials for the CVD preparation. Figure 1 shows the overall experiment setup. An Ar-gas supplier and a vacuum pump are mounted on left and right side of a two-zone tube furnace respectively, which provides the growth environment for CVD preparation of 2D materials.

For the in-situ DRS measurements, a home-built setup is implemented above the tube furnace. A light beam is obtained by a broadband light source that is either a xenon lamp or a tungsten-halogen lamp. It enters into the furnace via a quartz window after the light beam is collimated by the lens (L1) and focused by the mirror (M1). The reflected light beam also passes through the quartz window. Following, the mirror (M2) and the lens (L2) gather the reflected light beam, which is transmitted into the fiber. Additionally, the lenses (L1 and L2) and mirrors (M1 and M2) are installed on an optical adjustable bracket to achieve their four-axial (x, y, z, *θ*) movement. A high-resolution spectrometer (QE Pro, Ocean Optics, Dunedin, FL, USA) connected to the fiber is acted as a spectra collector. The computer averages 1600 successive spectral data at a time to reduce the statistical noise of spectra, which spends around 120 s. We take such a set of cumulative data as one spectrum acquired during the in-situ experiment. The overall setup achieves an in-situ observation without interrupting CVD preparation, which ensures the growth and characterization of 2D materials at the same time.

High temperature and a wide range of temperature variations are needed during the CVD experiment, which lead to the thermal deformation of mechanical structures and changes in the optical coefficient of the substrate. Thus, the general DRS (Formula (1)) can not satisfy the requirements of measurement accuracy during CVD growth. It is assumed that the variations of light intensity induced by thermal deformation and changes in substrate optical coefficient are repeatable. Here, the new DR spectra suitable for variable temperature environment are calculated by the equation as follow:

(2) $$\frac{\Delta R}{R}=\frac{R_{S}(T_{i})-R_{0}(T_{i})}{R_{0}(T_{i})},$$

where *R*_0_*(T_i_)* and *R_S_(T_i_)* denote the reflectivity of the bare substrate without MoS_2_ growth and the one of the substrate with the MoS_2_ growth at the same temperature *T_i_*, respectively. Before the 2D materials growth experiment, we have heated the bare substrate in the range of the preparation temperature to obtain the reflectivity of the bare substrate with the same time interval, temperature and airflow. The new DRS signal *∆R/R* thus inhibits the measurement error caused by temperature and reveals the optical properties of the surface on a substrate.

## 3. Results and Discussion

### 3.1. Ex-Situ Characterization

**Figure 2:**
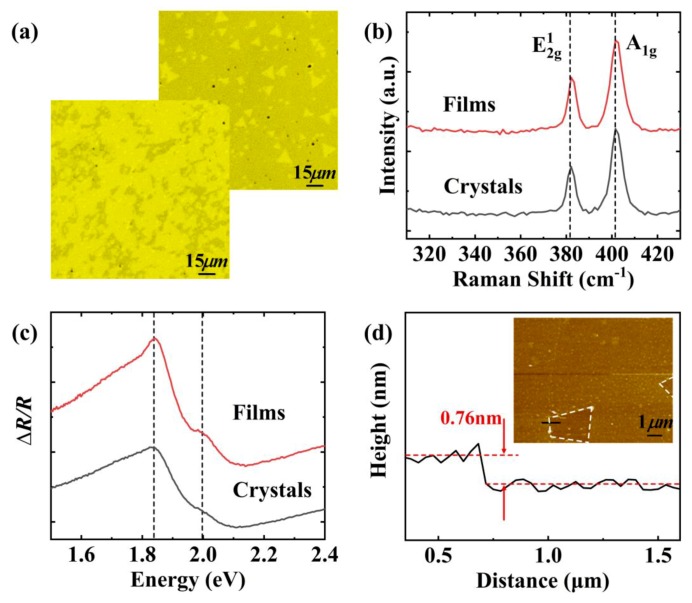
(**a**) Optical images of MoS_2_ thin films (front) and independent triangular crystals (back) on two different regions of the same SiO_2_/Si substrate; (**b**) Raman spectra of MoS_2_ films and independent triangular crystals; (**c**) Ex-situ differential reflectance spectroscopy (DRS) obtained on an optical platform at room temperature; (**d**) The height profile of MoS_2_. The inset shows an atomic force microscopy (AFM) image. The black solid line is the measurement route of a probe. The darker region is the area without MoS_2_, which marked with white dashed lines.

The MoS_2_ sample was prepared by atmospheric pressure CVD in a two-zone tube furnace, which was characterized ex-situ by optical microscopy, Raman spectroscopy, DRS and AFM. After cooling down to the room temperature, optical microscopy was performed to observe the distribution and morphology of MoS_2_ on the SiO_2_/Si substrate. Figure 2a displays the optical images at different regions in the same sample. By the optical contrast, it is found that most regions of SiO_2_/Si surfaces are covered with MoS_2_ thin films. The optical contrast of the sample is not uniform, which indicates the substrate is not completely covered with MoS_2_ and the surfaces are not flat as shown in the front image from Figure 2a. Moreover, there are some independent triangular MoS_2_ crystals in the edge regions of the substrate far from the molybdenum source. The size of independent triangular crystals is approximately 10 µm shown in Figure 2a back image. It implies that the substrate was covered with MoS_2_ in different states.

We employed Raman spectroscopy to characterize the sample structure at room temperature. Raman spectra of MoS_2_ sample are plotted in Figure 2b. The excitation wavelength is 532 nm. There are two characteristic peaks of MoS_2_ called E^1^_2g_ and A_1g_ in the spectra. The peak E^1^_2g_ at 382.1 cm^−1^ represents the in-plane reverse vibration mode of S and Mo atoms [21]. And another peak A_1g_ at 401.6 cm^-1^ is attributed to the out-of-plane vertical vibration mode of S atoms [21]. In particular, the wavenumber difference of two peaks is approximately 19.5 cm^−1^, which indicates that the MoS_2_ structures of films and independent triangular crystals are monolayer structures [32].

In order to study the optical absorption of MoS_2_ and as a reference for in-situ spectra, ex-situ DR spectra were performed at room temperature. As shown in Figure 2c, ex-situ DR spectra were measured on an optical platform. The absorption peaks at 1.84 eV and 2.0 eV correspond to characteristic peaks A and B, respectively. They are induced by exciton transitions at the point K of the Brillouin zone. The energy interval between two exciton peaks is approximately 0.16 eV, owning to the splitting of valence bands at K point as a result of spin-orbit interaction [33].

AFM was selected to confirm the thickness and surface topography. Figure 2d presents the height profile across the edge of the MoS_2_ thin film. The inset in Figure 2d shows an AFM image. The height profile was measured along the black solid line in the AFM image. It displays a high gradient of approximately 0.76 nm, which consists of the thickness of monolayer MoS_2_ [34]. Therefore, the AFM result also agrees well with the DRS and Raman characterizations.

### 3.2. In-Situ DRS

**Figure 3:**
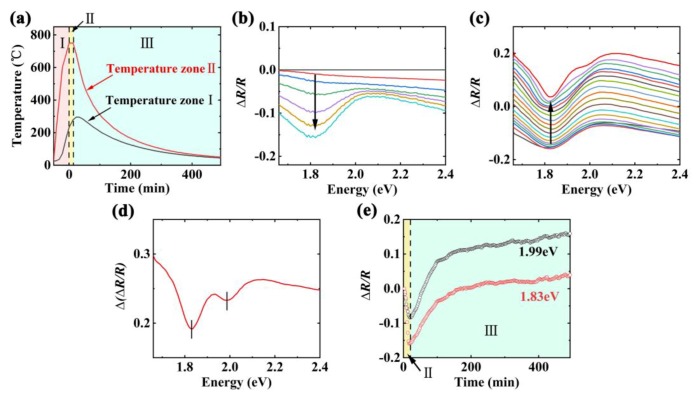
(**a**) The temperature curves of temperature-zone І (black line) and ІІ (red line) during the chemical vapor deposition (CVD) growth, respectively. The preparation process is divided into the stage І, stage ІІ, and stage ІІІ. (**b**) In-situ differential reflectance (DR) spectra recorded in the stage ІІ during CVD preparation of monolayer MoS_2_ on a SiO_2_/Si substrate. The time interval between successive spectra is 120 s. The black arrow indicates the direction of spectral change at 1.83 eV. (**c**) In-situ DR spectra recorded in the stage ІІІ. The temperature interval is 40 °C and the temperature range is 730–50 °C. The black arrow indicates the direction of temperature reduction. (**d**) The increment of DR spectra during the stage ІІІ. (**e**) The intensities of DR signals at 1.83 eV and 1.99 eV as a function of time, respectively.

CVD growth process of MoS_2_ introduced in the experimental section was observed using in-situ DRS. According to the temperature in a two-zone tube furnace, the preparation process of MoS_2_ can be separated into three sequential stages in Figure 3a. The stage І (the heating-up stage) began at room temperature and stopped at 750 °C of the temperature zone ІІ. Almost no MoS_2_ film on the substrate was observed in this stage. For the stage ІІ (the thermostatic stage) at the temperature of 750 °C, MoS_2_ thin film began to deposit on the substrate with the increased lateral size. During the stage ІІІ (the cooling-down stage), the growth of MoS_2_ thin film slowed down and ended gradually. The temperature dropped to room temperature.

The spectral evolution of reflected light from the substrate during the thermostatic stage is displayed in Figure 3b. The time interval between successive spectra is 120 s. The formation and evolution of the absorption peak at 1.83 eV are observed along the arrow direction. It can be noted that the absorption peak is in accordance with the optical characteristic peak A of monolayer MoS_2_. This observation implies that the reaction between molybdenum trioxide and sulfur has occurred and MoS_2_ has begun to grow on a SiO_2_/Si substrate during the thermostatic stage. In addition, the DR intensity at 1.83 eV of this stage decreases continuously. It could be inferred that DR intensity correlates with the increase of growth area and absorption for MoS_2_ sample under the constant temperature.

Figure 3c exhibits DR spectra of MoS_2_ on a SiO_2_/Si substrate during the cooling-down stage in which the development of spectral intensity and shape is displayed. The successive spectra were recorded with the temperature interval of 40 °C. The temperature range is 730–50 °C. Each spectrum we collected is the average of cumulative spectra over a short period of time (around 120 s), which represents the average state of MoS_2_ during each time interval. Different from the thermostatic stage, the spectral amplitudes of the cooling-down stage rise regularly. This finding indicates that the amplitude is related to variation of temperature. Apparently, there are two negative peaks around 1.83 eV and 1.99 eV appearing during this process. The two peaks are consistent with the characteristic peaks A and B of monolayer MoS_2_, respectively. This observation indicates that monolayer MoS_2_ has grown on the SiO_2_/Si substrate, agreeing with the results of ex-situ characterizations. The width of the absorption peak at approximately 1.83 eV becomes narrower when the temperature decreases. This can be attributed to the widening of the peak induced by high temperature, which is in accordance with the previous reports [35,36,37].

In order to further detect the increment of DR spectra during the cooling-down stage, the *∆(∆R/R)* spectrum is shown in Figure 3d. It can be clearly noted that two prominent absorption peaks of MoS_2_ are observed by the increment of DR signals. The peak A at 1.83 eV is relatively narrow, which is generated by exciton transition of electron-hole recombination. Another peak B induced by exciton transition at the lower valence band of K point is also obvious at 1.99 eV. The appearance of two peaks is a clear growth signature for monolayer MoS_2_. It should be pointed out that the positions of absorption peaks have red shifts in this stage, which are compared with the ex-situ DR spectra at room temperature. We can attribute it to the high temperature of the substrate. This observation is consistent with the previous report that high temperature could cause the red shifts of peaks [27]. In addition, the DT increments of characteristic peaks A and B in the [27] are very weak (less than 0.1). Both the front surface and the back surface of a substrate were covered by monolayer MoS_2_. However, the DR increment of peak A and B is approximately 0.19 and 0.23 respectively. Monolayer MoS_2_ was only deposited on the front surface of a substrate and didn’t completely cover the front surface. The signal increases more obvious from reflectance measurements compared with that from transmittance measurements, which indicates that the reflectance measurement has a higher sensitivity for 2D materials.

Furthermore, the DR intensity at 1.83 eV and 1.99 eV are respectively plotted as a function of time in Figure 3e. This is helpful to understand the detailed evolution of the two peaks. We take the initial time of the thermostatic stage as the founding moment. As shown in Figure 3e, the values of *∆R/R* drop during the growth of the thermostatic stage and then the values rise obviously in the cooling-down stage. This result is the same as the tendency shown in Figure 3b,c. It displays that the growth of MoS_2_ is closely related to temperature.

## 4. Conclusions

In summary, the growth of MoS_2_ prepared by CVD on a SiO_2_/Si substrate was in-situ characterized by DR spectra. An in-situ DRS setup monitoring CVD growth of 2D materials was established in order to achieve this aim. The process of the growth and cooling down of monolayer MoS_2_ was investigated by the in-situ DRS. An obvious optical feature of MoS_2_ in the in-situ DR spectra was observed during thermostatic growth. DR spectra of the cooling-down stage showed the evolution of the MoS_2_ optical characteristics, indicating the formation of monolayer MoS_2_. Moreover, ex-situ DRS also exhibited the characteristic peaks of monolayer MoS_2_. It agrees well with other ex-situ characterization results such as optical microscopy, Raman spectroscopy and AFM, which confirmed the monolayer structure of MoS_2_. The results emphasize that DRS is effective and has a high sensitivity for in-situ observations of 2D TMDCs during the CVD growth.
